# Evaluation of the Antimicrobial Effect of Mineral Trioxide Aggregate Mixed with Fluorohydroxyapatite against *E. faecalis* In Vitro

**DOI:** 10.1155/2021/6318690

**Published:** 2021-11-24

**Authors:** Behnam Bolhari, Aidin Sooratgar, Maryam Pourhajibagher, Nazanin Chitsaz, Iman Hamraz

**Affiliations:** ^1^Department of Endodontics, School of Dentistry, Tehran University of Medical Sciences (TUMS), Tehran, Iran; ^2^Department of Endodontics, Tehran University of Medical Sciences (TUMS), International Campus, Tehran, Iran; ^3^Dental Research Center, Dentistry Research Institute, Tehran University of Medical Sciences (TUMS), Tehran, Iran; ^4^Department of Biostatistics, Faculty of Medicine, Arak University of Medical Sciences, Arak, Iran

## Abstract

*Enterococcus faecalis* is the dominant microorganism in chronic apical periodontitis. It is more resistant to local antiseptic agents than other endodontic microorganisms. Currently, mineral trioxide aggregate (MTA) is considered as an ideal material in many endodontic procedures. Some studies have shown that MTA has good antibacterial activity against *E. faecalis*. However, some studies have investigated the effect of incorporating some materials into MTA on its antibacterial activity against *E. faecalis*. No study has evaluated the effect of incorporating fluorohydroxyapatite nanoparticles (nano-FHA) on the antimicrobial activity of MTA. Therefore, the present study evaluated the antimicrobial effect of MTA mixed with nano-FHA on *E. faecalis* in vitro. The study was carried out on 18 samples in three groups: pure MTA, MTA mixed with 10 wt% of nano-FHA, and MTA mixed with 15 wt% of nan-FHA. The effect of nano-FHA on the antibacterial activity of MTA on *E. faecalis* was evaluated by evaluating the growth inhibition zone around each sample. The antimicrobial effect of samples on inhibiting *E. faecalis* biofilm formation and inhibiting microbial growth of *E. faecalis* in the planktonic phase was evaluated by disk agar diffusion (DAD), biofilm inhibition assay (BIA), and direct contact assay (DCA) tests, respectively. All the above tests were analyzed after 24 and 72 hours. Factorial designs were used for statistical analyses. Tukey tests were used for two-by-two comparisons. All the statistical analyses were carried out with SPSS 26. DAD results showed no formation of the growth inhibition zone in all the samples after 24 and 72 hours. The microbial colony counts in the BIA and DCA tests in the groups modified with FHA nanoparticles were significantly lower than the pure MTA group (*P* < 0.05). The microbial colony counts increased in all the groups over time (*P* < 0.05). Incorporating nano-FHA into MTA improved the antimicrobial activity of MTA against *E. faecalis* compared to pure MTA. The highest antimicrobial activity was achieved after incorporating 15 wt% of nano-FHA into MTA at the 72-hour interval.

## 1. Introduction

The main reasons for pulpal and periapical diseases and failure of endodontic treatments are microorganisms [1, 2]. Therefore, eliminating microorganisms by instrumentation, irrigation, and intracanal medications during root canal treatment is necessary [3]. However, it is not possible to completely clean root canals from bacteria and their products due to the complexity of the root canal system. Therefore, proper obturation of the entire root canals or the perforation area has a vital role in preventing reinfection by residual microorganisms due to the antimicrobial activity of sealing materials [4, 5]. MTA is composed of 80% Portland cement and 20% bismuth oxide (radiopacifier). The cement is made up of calcium, silicon, and aluminium. The main constituent phases are tricalcium and dicalcium silicate and tricalcium aluminate. There are gray and white forms of MTA. The phase tetracalcium aluminoferrite is absent in white MTA [6]. Currently, MTA is considered as an ideal material in many endodontic processes, including pulp capping, pulpotomy, apexogenesis/apexification, root resorption, lateral or furcal perforation repair, and retrograde obturation [7] because this material induces the growth of cementum and PDL, resulting in proper periodontal healing [8]. However, MTA has some inherent disadvantages, including difficult handling, long setting time, and discoloration potential [9]. Since the introduction of ProRoot MTA in 1998, new products have been produced based on MTA, including MTA Angelus and Endocem MTA, to overcome these deficiencies by modifying the composition or concentration of each material [7]. MTA Angelus (Angelus, Londrina, PR, Brazil) is a biocompatible material produced for bone repair procedures and has been compared with Portland cement due to similarities in their chemical structure and tissue response [10, 11].

Recently, several studies have evaluated the effect of MTA on microorganisms related to endodontic diseases [10, 12, 13]. *Enterococci*, *Actinomycetes*, *Propionibacterium*, yeasts, and *Streptococci* are some of the microorganisms isolated from the infected root canals [14]. However, many clinicians believe that *E. faecalis* is the dominant microorganism in chronic apical periodontitis (retreatment cases) [15, 16]. This microorganism is a Gram-positive [1], facultative anaerobic, bacterial species [17] that can survive without water and nutrients for several months [18]. It is also more resistant to local disinfectants than other endodontic microorganisms [19].

Some studies, including that by Morgental et al. (2011), have shown that MTA does not exhibit significant antibacterial activity against *E. faecalis* after setting [20]. Therefore, several studies have evaluated the effect of incorporating some materials, including 2% chlorhexidine (CHX) gluconate, tetracycline powder, and calcium hydroxide, on the antibacterial activity of MTA against *E. faecalis*, concluding that incorporating CHX into MTA increased its antimicrobial activity [14].

Hydroxyapatite (HA) is a mineral agent with a chemical structure of Ca_10_(PO_4_)_6_(OH)_2_ that comprises 60–70% of the mineral matrix of the bone and has high bioactivity and biocompatibility [21, 22]. This material has many applications in biomedicine, especially in orthopedics and dentistry [23]. Incorporating fluorine into hydroxyapatite results in the synthesis of fluorohydroxyapatite (FHA: Ca_5_(PO_4_)_3_(OH)_1-x_F_x_), which is more stable chemically than hydroxyapatite, with low solubility [3]. Some studies have even shown that FHA has higher antimicrobial activity than pure HA [24].

Promising characteristics of FHA have made it a good material in orthopedics and dentistry [25]. However, limited reports are available on incorporating this material into other commonly used materials in dentistry, and no study is available on the effect of this material on the antimicrobial activity of MTA. Therefore, the present study aimed to evaluate the antimicrobial activity of MTA mixed with fluorohydroxyapatite against *E. faecalis* in vitro.

## 2. Materials and Methods

### 2.1. The Synthesis of Fluorohydroxyapatite (Nano-FHA)

Four-water calcium nitrate solution was prepared with 0.3 M concentration and placed on a magnetic mixer. The electrode of the pH meter (WTW, Germany) was placed within the reaction container to measure the pH of the container continuously. Then, a 1 M solution of sodium hydroxide was added to the solution until the pH was stabilized between 10 and 11. Subsequently, 0.18 M ammonium dihydrogen phosphate and 0.18 M sodium fluoride were added to the calcium nitrate solution dropwise. During the reaction, the pH was kept between 10 and 11 by adding NaOH.

To separate fluorohydroxyapatite, the resulting solution was cleared through a filter, and the remaining liquid was transferred to a centrifuge container. The liquid was washed with water and finally with acetone after several times of centrifugation. The gels achieved from the centrifugation process were dried by freeze-drying. The dried powder was sintered in an oven at 600°C for 6 hours. The heat increase rate in the oven was 2°C/min [26].

### 2.2. Preparation of Samples

The pilot was done to determine the percentages of adding FHA to MTA. Adding percentages lower than 10% did not give any special properties to the material. Adding percentages greater than 15% made difficult handling of the material.

**Figure 1:**
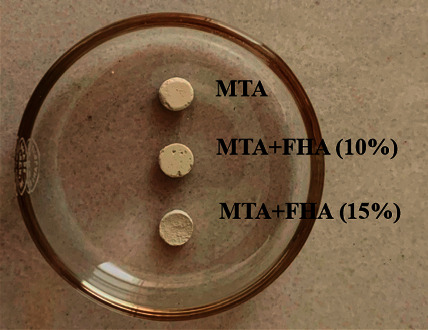
The samples were prepared in three study groups.

To prepare the test groups, the commercial MTA powder was mixed with 10 and 15 wt% of nano-FHA as follows: nano-FHA and commercial MTA powders were weighed with an accurate weighing machine and divided into equal parts. Then, each equal pat of nano-FHA powder (for one group with a 10 wt% of the final powder and for one group with a 15 wt% of the final powder) was manually mixed with MTA Angelus powder for three minutes, followed by mixing in an amalgamator (Golden Amalgamator, China), to achieve a uniform distribution of particles. Therefore, three groups were prepared as follows (Figure 1):

1. MTA
2. MTA + FHA (10 wt%)
3. MTA + FHA (15 wt%)

### 2.3. Antimicrobial Tests

The effect of nano-FHA on the antimicrobial activity of MTA against *E. faecalis* was evaluated by evaluating the growth inhibition zone around each sample. In addition, the antimicrobial effect of the samples on inhibiting *E. faecalis* biofilm formation and growth inhibition of *E. faecalis* in the planktonic phase was evaluated with DAD, BIA, and DCA tests. All the above tests were analyzed after 24 and 72 hours.

### 2.4. Disk Agar Diffusion Test

An 0.5 McFarland suspension (1.5 × 10^8^ CFU/mL) was prepared from *E. faecalis* bacterial species in the BHI (brain heart infusion) broth (Merck, Darmstadt, Germany) and seeded on the BHI agar medium [27]. After the culturing procedure, the prepared disks were placed on the surface of the culture medium inoculated with microorganisms at a distance of 2 mm from each other, and the plates were incubated at 37°C for 24 hours [28]. The results were reported by measuring the diameters of the growth inhibition zones with a ruler [29].

### 2.5. Biofilm Inhibition Test

To carry out this test, first, the disks containing different concentrations of nano-FHA were placed in 48-well microplates. Then, 1 mL of the microbial suspension at 0.5 McFarland concentration (1.5 × 10^8^ CFU/mL) was added to each well. Subsequently, the 48-well microplates were incubated at 37°C for 24 and 72 hours [28]. After these time intervals, each disk was rinsed in tubes containing 1 mL of sterile normal saline solution for 1 minute. A sonicator was used to detach microbial biofilms formed on the disk surfaces. Then, the microbial suspensions achieved were diluted and cultured in a BHI agar medium. The microbial colony counts were determined based on previous studies [30].

### 2.6. Direct Contact Assay

To carry out this test, the disks with different concentrations were placed in tubes containing 500 *µ*L of the microbial suspension with a concentration of 1.5 × 10^8^ CFU/mL and incubated at 37°C for 24 and 72 hours. After these time intervals, 10 *µ*L of the content of each tube was transferred into the BHI agar medium and spread-cultured on the surface of the culture mediums. The colony counts were determined similar to previous studies and reported in CFU/mm^2^ [28].

### 2.7. Statistical Analysis

The descriptive data of the study were reported using descriptive statistics, including means, minimums, maximums, and standard deviations. Factorial designs were used to compare microbial tests, and the tests were reported three times considering the factors of time and the type of the microbial test. Tukey tests were used for two-by-two comparisons. SPSS 26 was used for all the statistical analyses.

## 3. Results

### 3.1. The Results of the Disk Agar Diffusion Antimicrobial Test

**Figure 2:**
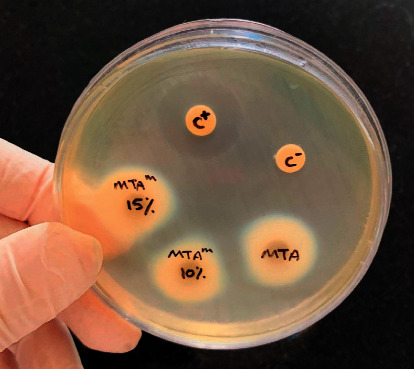
Growth inhibition zones in the samples after 24 hours (positive control, 0.2% CHX; negative control, water).

This test showed the absence of growth inhibition zones in all the samples after 24 hours (Figure 2).

### 3.2. The Results of Biofilm Inhibition and Direct Contact Assay Antimicrobial Tests

Factorial designs were used to evaluate the antimicrobial effect of MTA-FHA on *E. faecalis* at 24- and 72-hour intervals.  Table 1 presents the descriptive statistics (colony counts) in the study groups at the above intervals based on DCA and BIA antimicrobial tests.

According to Table 1, the DCA test showed a decrease in microbial biofilm formation on the disk surfaces with an increase in the percentage of FHA incorporated into MTA. After 24 hours, the microbial biofilm in the MTA group modified with 10% FHA decreased 24.47% compared to the pure MTA group (*P* < 0.05); in the MTA group modified with 15% FHA, it decreased 45.57% compared to the pure MTA group (*P* < 0.05). After 72 hours, the microbial biofilm in the MTA group modified with 10% FHA decreased 18.49% compared to the pure MTA group (*P* < 0.05); in the MTA group modified with 15% FHA, it decreased 43.7% compared to the pure MTA group (*P* < 0.05).

In all the study groups, biofilm formation increased over time after 72 hours compared to the 24-hour interval (the pure MTA group: 32.2% increase, the MTA-10% FHA group: 42.67% increase, and the MTA-15% FHA group: 36.74% increase) (*P* < 0.05).

According to Table 2, the results of the BIA test were similar to those of the DCA test: an increase in the percentage of FHA incorporated into MTA decreased microbial biofilm formation (*P* < 0.05) (at both 24- and 72-hour intervals). The microbial biofilm formation increased after 72 hours compared to the 24-hour interval in all the study groups (*P* < 0.05).

Table 3 presents the results of two-by-two comparisons of the groups with post hoc Tukey tests. Based on the results, in the DCA test, the greatest differences were observed between the MTA and MTA-15% FHA groups (*P* < 0.001), followed by the MTA-10% FHA and MTA-15% FHA groups (*P*=0.001). In the BIA test too, the greatest differences were observed between the MTA and MTA-15% FHA groups (*P* < 0.001), followed by the MTA and MTA-10% FHA groups (*P*=0.001).

**Figure 3:**
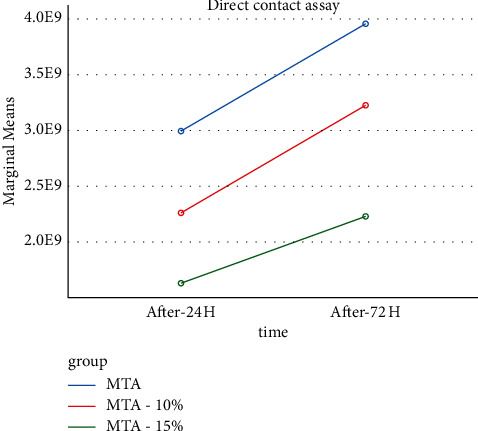
The linear graph of the comparison of the groups with the DCA test.

**Figure 4:**
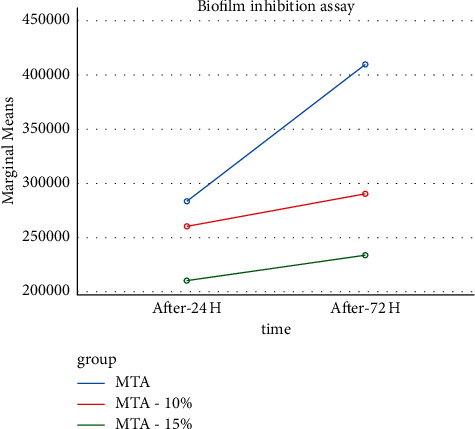
The linear graph of the comparison of the groups with the BIA test.

Figures 3 and 4 show the linear comparison of the groups and time intervals in the DCA and BIA groups.

According to Figure 3, which shows the linear graph of the DCA test in the three study groups, microbial biofilm formation increased over time in all these groups. The MTA-15% FHA group exhibited the least microbial biofilms, and the MTA group exhibited the highest microbial biofilm formation at 24- and 72-hour intervals.

According to Figure 4, which shows the liner graph of the BIA test in the three study groups at 24- and 72-hour intervals, the microbial biofilm formation in the MTA-15% FHA group was less than the two other groups. Microbial biofilm formation increased over time in all the three groups, with a steeper increase in the MTA group than in the two other groups.

## 4. Discussion

After root canal obturation, a proper seal is necessary to ensure root canal treatment success, especially after eliminating *E. faecalis* and cleaning the root canal with chemomechanical methods [1]. Although various materials are used to obturate the root canals, none is absolutely ideal [31], and many materials might not provide a hermetic seal. Therefore, it has been suggested that these materials should prevent bacterial growth [32] and have antibacterial properties.

Several studies have investigated the antimicrobial activity of MTA against *E. faecalis*, with contradictory results. For example, Prathia et al. (2019) showed that MTA has a better antimicrobial effect on this microorganism than calcium hydroxide sealer [17]. In a study by Usman et al. (2017), using the direct contact test, the MTA sealer exhibited better antimicrobial activity than a bioceramic sealer seven days after mixing [1]. However, Kim et al. (2015) reported that MTA Angelus and ProRoot MTA could not prevent the growth of *E. faecalis* [7]. Prathia et al. (2019) and Usman et al. (2017) used the DCA test. Kim et al. (2015) used the DAD test. In the present study, we used DAD, BIA, and DCA tests to evaluate antibacterial properties of the materials. Some studies have hypothesized that MTA releases Ca(OH)_2_ upon contact with tissue fluid. Therefore, a high pH value results in continuous antimicrobial activity up to seven days after mixing [17, 33, 34]. However, some other reports have reported that the antibacterial activity cannot be explained only based on the pH value [7] because, under clinical conditions, the high pH value of MTA cannot be maintained due to the buffering capacity of dentin [7]. In addition, *E. faecalis* has a proton pump that helps decrease intracellular pH [35]. In the present study, no growth inhibition zones were detected after 24 and 72 hours, which might be related to the above explanation.

To improve the antibacterial activity of MTA against *E. faecalis*, researchers have investigated incorporating various materials into its structure, including 0.2% CHX, tetracycline, and calcium hydroxide [14]. However, the effect of adding FHA to antimicrobial properties of MTA has not been studied yet. Since there is no similar study to compare the results, further studies are needed.

Only one study evaluated the effect of incorporating this material (nano-FHA) on the antimicrobial activity of the AH26 epoxy resin sealer against *E. faecalis* and *Streptococcus mitis*, with the results indicating its positive antibacterial effect compared to the control group [36].

In the present study too, incorporating this material into MTA positively affected its antibacterial effects on *E. faecalis*. After 24 and 72 hours, the microbial colony counts in the groups modified with nano-FHA were significantly lower than those in the pure MTA group. An increase in the concentration of nano-FHA improved its antibacterial activity.

Wang et al. (2017) carried out a review and described different mechanisms for the antibacterial properties of nanoparticles [37], including oxidative stress induction, the release of metallic ions, and nonoxidative mechanisms [37]. On the other hand, *E. faecalis* is a Gram-positive bacterial species, and many studies have shown that nanoparticles exhibit greater antimicrobial activity against Gram-positive bacteria than Gram-negative bacteria [37] because the cell wall of Gram-positive bacteria consists of a thin layer of peptidoglycan and teichoic acid, with many pores allowing the penetration of extrinsic molecules, resulting in cell membrane damage and cellular death [37]. In addition, Gram-positive bacteria have a high negative charge on their cell wall that can adsorb nanoparticles [38].

In addition to the mechanisms mentioned above for the antibacterial properties of nanoparticles, fluoride can disrupt the metabolism and growth of oral bacteria directly and indirectly through other complex mechanisms too [39], including disrupting the glycolytic pathway by inhibiting enolase and metalloenzyme, direct inhibition of H+/APPase, decreasing cellular content of peptidoglycans, and interfering with the synthesis of glycogen [39–41]. The results of the studies above might explain the results of the present study.

## 5. Conclusion

The incorporation of nano-FHA into MTA increased its antimicrobial activity against *E. faecalis* compared to pure MTA. Incorporating 10 and 15 wt% of FHA into MTA significantly increased its antimicrobial activity. In addition, the antimicrobial activity was higher at the 72-hour interval compared to the 24-hour interval. The highest antibacterial activity was observed after incorporating 15 wt% of FHA into MTA at the 72-hour interval. It is suggested that future studies evaluate other properties and incorporation of other weight percentages of nanoparticles into MTA.

## Figures and Tables

**Table 1:** The means and standard deviations of colony counts (CFU/mL) based on the DCA test in the study groups.

Microbial test	Groups	After 24 hours				After 72 hours			
		Mean	SD	Min	Max	Mean	SD	Min	Max
DCA	MTA	30 × 10^8^	2.64 × 10^8^	28 × 10^8^	33 × 10^8^	39.66 × 10^8^	3.05 × 10^8^	37 × 10^8^	43 × 10^8^
	MTA-10% FHA	22.66 × 10^8^	2.08 × 10^8^	21 × 10^8^	25 × 10^8^	32.33 × 10^8^	3.21 × 10^8^	30 × 10^8^	36 × 10^8^
	MTA-15% FHA	16.33 × 10^8^	2.08 × 10^8^	14 × 10^8^	18 × 10^8^	22.33 × 10^8^	3.05 × 10^8^	19 × 10^8^	25 × 10^8^

**Table 2:** The means and standard deviations of colony counts (CFU/mL) based on the BIA test in the study groups.

Microbial test	Groups	After 24 hours				After 72 hours			
		Mean	SD	Min	Max	Mean	SD	Min	Max
BIA	MTA	28.33 × 10^4^	2.51 × 10^4^	26 × 10^4^	31 × 10^4^	41 × 10^4^	4.58 × 10^4^	36 × 10^4^	45 × 10^4^
	MTA-10% FHA	26 × 10^4^	1.73 × 10^4^	24 × 10^4^	27 × 10^4^	29 × 10^4^	2.64 × 10^4^	26 × 10^4^	31 × 10^4^
	MTA-15% FHA	21 × 10^4^	2.64 × 10^4^	18 × 10^4^	23 × 10^4^	23.33 × 10^4^	3.51 × 10^4^	20 × 10^4^	27 × 10^4^

**Table 3:** Comparison of the study groups based on the DCA and BIA tests.

	Groups		M.D	*P* value	LB	HB
Antimicrobial tests	MTA	MTA-10% FHA	7.33 × 10^8^	0.001	3.13 × 10^8^	11.53 × 10^8^
		MTA-15% FHA	15.50 × 10^8^	<0.001	11.29 × 10^8^	19.70 × 10^8^
	MTA-10% FHA	MTA-15% FHA	8.16 × 10^8^	0.001	3.96 × 10^8^	12.36 × 10^8^
BIA	MTA	MTA-10% FHA	7.16 × 10^4^	0.004	2.43 × 10^4^	11.90 × 10^4^
		MTA-15% FHA	12.50 × 10^4^	<0.001	7.76 × 10^4^	17.23 × 10^4^
	MTA-10% FHA	MTA-15% FHA	5.33 × 10^4^	0.027	0.59 × 10^4^	10.06 × 10^4^

## Data Availability

The data used to support the findings of this study are included within the article and are available from the corresponding author upon request.
